# Is Deafness Increasing?

**Published:** 1882-05

**Authors:** 


Is Deafness Increasing ?—The hearing of school children has received considerable attention recently. Dr. Weil of Stuttgart has in the last two years examined 4,500 children, thirty per cent of whom were found to hear imperfectly with one ear. Dr. Reichard of Riga examined 1,055 children and found twenty-two per cent and a fraction with defective hearing of one or both ears. Dr. Sexton of this city and Dr. C. F. Percivall of Baltimore have made similar investigations and have arrived at like conclusions. The disquieting question arises whether the human race is becoming deaf as well as near-sighted as civilization increases ?

